# A Pituitary Carcinoma Patient With Cerebrospinal Fluid Dissemination Showing a Good Response to Temozolomide Combined With Whole-Brain and Spinal Cord Radiotherapy: A Case Report and Literature Review

**DOI:** 10.3389/fonc.2022.890458

**Published:** 2022-07-12

**Authors:** Peng Du, Xuefan Wu, Kun Lv, Ji Xiong, Daoying Geng

**Affiliations:** ^1^ Department of Radiology, Huashan Hospital, Fudan University, Shanghai, China; ^2^ Center for Shanghai Intelligent Imaging for Critical Brain Diseases Engineering and Technology Research, Huashan Hospital, Fudan University, Shanghai, China; ^3^ Department of Radiology, Shanghai Gamma Hospital, Huashan Hospital, Fudan University, Shanghai, China; ^4^ Department of Pathology, Huashan Hospital, Fudan University, Shanghai, China

**Keywords:** whole-brain and spinal cord radiotherapy, pituitary carcinoma, pituitary tumor, pituitary adenoma, temozolomide, case report

## Abstract

Pituitary carcinoma (PC) is extremely rare, with its incidence only accounting for 0.1%-0.2% of pituitary tumor (PT). Existing histological features, including invasiveness, cellular pleomorphism, nuclear atypia, mitosis, necrosis, etc., can be observed in pituitary adenoma (PA), invasive PA (IPA) and PC. Invasion is not the basis for the diagnosis of PC. The diagnosis of PC is often determined after the metastases are found, hence early diagnosis is extraordinarily difficult. Owing to the conventional treatment for PC may not be effective, a large portion of patients survived less than one year after diagnosis. Therefore, it is of great significance to find an efficacious treatment for PC. We report a rare case of sparsely granulated somatotroph carcinoma with cerebrospinal fluid dissemination showing a favorable treatment response to temozolomide (TMZ) combined with whole-brain and spinal cord radiotherapy.

## Introduction

PC is extremely rare, and its diagnosis is mainly based on the presence of primary adenohypophyseal tumor in the sellar region (SR) and metastases of adenohypophysis origin in the brain, spinal cord, or other distant organs outside the SR (1, 2). Existing histological features, including invasiveness, cellular pleomorphism, nuclear atypia, mitosis, necrosis, etc., can be observed in PA, IPA and PC (3). Invasion is not the basis for the diagnosis of PC. Many IPAs invade the surrounding structures of the SR, such as bone, dural mater, and cavernous sinus. Still, they are limited to the SR and cannot be defined as PC. The diagnosis of PC is determined only after the metastases are found. In addition, the clinical manifestations of PC are similar to PA and IPA, mainly due to the compression of the tumor on the surrounding tissue and symptoms caused by excessive hormone secretion, and it is challenging to identify PC from these non-specific clinical manifestations (4, 5). Thus, the diagnosis of PC is often delayed, and early diagnosis is extremely difficult. Owing to the conventional treatment for PC may not be effective, a large portion of patients survived less than one year after diagnosis (6). Therefore, it is of great significance to find an efficacious treatment for PC. We report a rare case of PC patient with cerebrospinal fluid dissemination showing a favorable treatment response to TMZ combined with whole-brain and spinal cord radiotherapy.

## Case description

**Figure 1:**
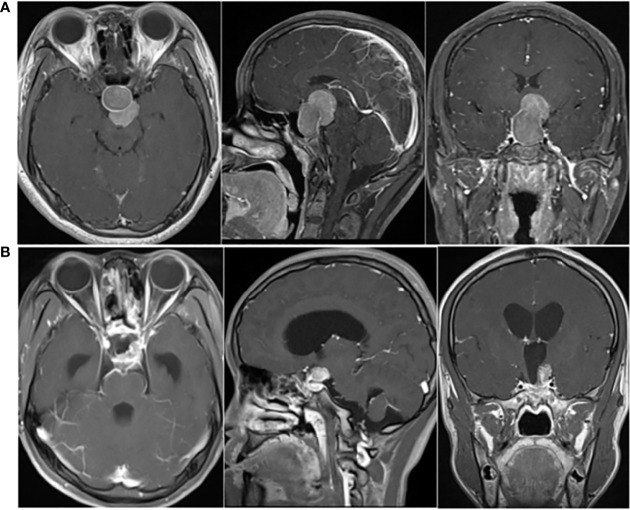
**(A)** Preoperative pituitary enhanced MRI (April 2016) showed: mass-shaped obvious enhanced signal could be seen in intrasellar and suprasellar area; the lesion extended upward to the suprasellar cistern, and the pituitary stalk was compressed, with the optic chiasm compressed and raised. **(B)** Postoperative pituitary enhanced MRI (October 2016) indicated: postoperative changes showed in the sellar region; abnormal enhancement of the upper left posterior pituitary appeared, with left cavernous sinus closely related.

A 21-year-old Chinese female had amenorrhea and lactation without obvious inducement, accompanied by dizziness, and blurred vision in the left eye since April 2016, with progressive aggravation of symptoms. Physical examination revealed that the patient had a hypertrophic nose, high cheekbones, bilateral fingers and toes that were thicker than peers. Laboratory test results showed: GH was 12.81 ng/mL (0.126-9.88 ng/mL), IGF-1 was 750.0 μg/L (115-358 μg/L), and no abnormality was found in PRL, FSH, LH, T3, T4, FT3, FT4, TSH and ACTH. The patient was in good health previously, without family history of tumor and other familial genetic diseases. Thus, the preliminary diagnosis was acromegaly. Subsequently, the patient underwent pituitary enhanced magnetic resonance imaging (MRI), and the results suggested: a tumor in the SR compressed the pituitary stalk, with the optic chiasm compressed and raised, and PA was considered (**Figure 1A**). A transnasal endoscopic sellar tumor resection was performed on August 17, 2016, and the postoperative pathological diagnosis was: PA [immunohistochemistry (IHC) suggested as somatotroph adenoma (SA)]. The patient’s vision improved after the operation.

The patient went to the hospital because of dizziness in October 2016. The GH level increased significantly (>50.00 ng/mL), and the pituitary enhanced MRI indicated: postoperative changes showed in the SR; abnormal enhancement of the upper left posterior pituitary appeared, with left cavernous sinus closely related, which was considered as postoperative residual of SA (**Figure 1B**). Therefore, adjuvant radiotherapy (ART) was recommended. The patient underwent ART from November 24 to December 29, 2016, at a dose of 40Gy/20Fx + 12Gy/6Fx.

**Figure 2:**
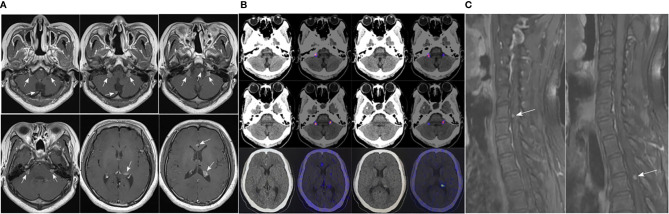
**(A)** Pituitary enhanced MRI in October 2020 indicated: multiple enhanced nodules appeared in bilateral auditory nerve area, pons, medulla oblongata and near cerebral falx; **(B)** 68Ga DOTA-TATE PET-CT in March 2021 showed: no abnormal increase in 68Ga DOTA-TATE uptake was seen in the low-density shadow in the sella turcica; 68Ga DOTA-TATE uptakes in multiple soft tissue nodules in the brain (obvious in the bilateral jugular foramen area, corpus callosum knee, left ventricular triangle, etc.) increased; **(C)** Before the patient underwent whole-brain radiotherapy, a spinal cord enhanced MRI was completed, which revealed abnormal enhanced nodules in the sixth cervical vertebrae and fourth thoracic vertebral plane of the spinal cord.

There were no obvious abnormalities during the regular follow-ups of pituitary enhanced MRI and GH level from April 2017 to April 2020. However, follow-up in October 2020 showed that GH was 14.29 ng/mL and pituitary enhanced MRI demonstrated multiple enhanced nodules (new lesions) in the bilateral auditory nerve area, pons, medulla oblongata and near cerebral falx (**Figure 2A**). The patient underwent 68Ga DOTA-TATE PET-CT on March 25, 2021 (**Figure 2B**), and the results suggested: 68Ga DOTA-TATE uptakes in multiple soft tissue nodules in the brain increased, and neoplastic lesions were considered.

**Figure 3:**
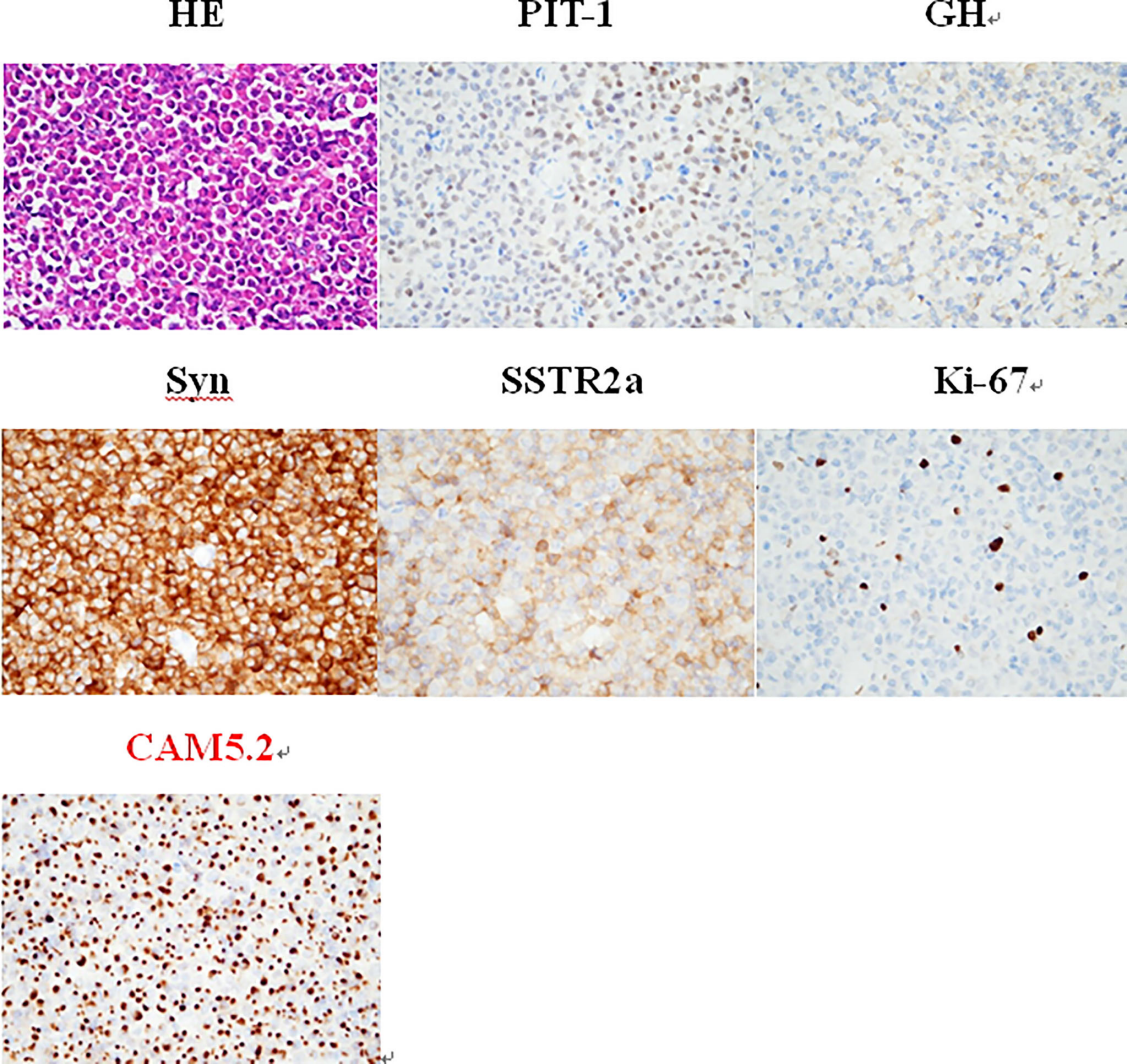
The IHC is positive for PiT-1, whereas keratin (CAM 5.2) reveals the presence of fibrous bodies in the cytoplasm. Therefore, the diagnosis of a somatotroph carcinoma of sparsely granulated type was established. HE staining (×400): small round nucleus tumor cells in a lamellar nest-like arrangement with rich cytoplasm and nucleus eccentricity, nucleus atypia with visible mitosis; IHC staining (×400): PIT-1 (+), GH (+), Syn (+), SSTR2a (+), Ki-67 (8%), and CAM5.2 (+).

To clarify the diagnosis and guide the subsequent treatment, the patient underwent resection of the right cerebellar vermis lesion on April 21, 2021. Intraoperatively, the right cerebellar hemisphere was seen near the median line with 1.5 cm × 1.0 cm tumor-like tissue on the cortical surface, adherent to the cortex, gray-red, tough texture, rich blood supply, and the tumor was resected en bloc along the border. The IHC results for keratin (CAM 5.2) revealed the presence of fibrous bodies in the cytoplasm. Also, taking into consideration the immunopositivity for PiT-1, the diagnosis of a carcinoma of sparsely granulated somatotroph type was established (**Figure 3**). Thus, the postoperative pathological diagnosis was: (cerebellum) sparsely granulated somatotroph carcinoma.

Thus, a treatment plan of taking TMZ orally combined with whole-brain radiotherapy was developed for the patient, with a dose of 150 mg/d of TMZ used every 28 days for 5 days and a whole-brain radiotherapy dose of 18Gy/10Fx + 21.6Gy/12Fx. Before the patient underwent whole-brain radiotherapy, a spinal cord enhanced MRI was completed, and abnormal enhanced nodules were found in the sixth cervical vertebrae and fourth thoracic vertebral plane of the spinal cord, which were considered as metastases (**Figure 2C**). Hence the treatment plan was adjusted to whole-brain and spinal cord radiotherapy, with a dose of 5.4Gy/3Fx for spinal cord.

**Figure 4:**
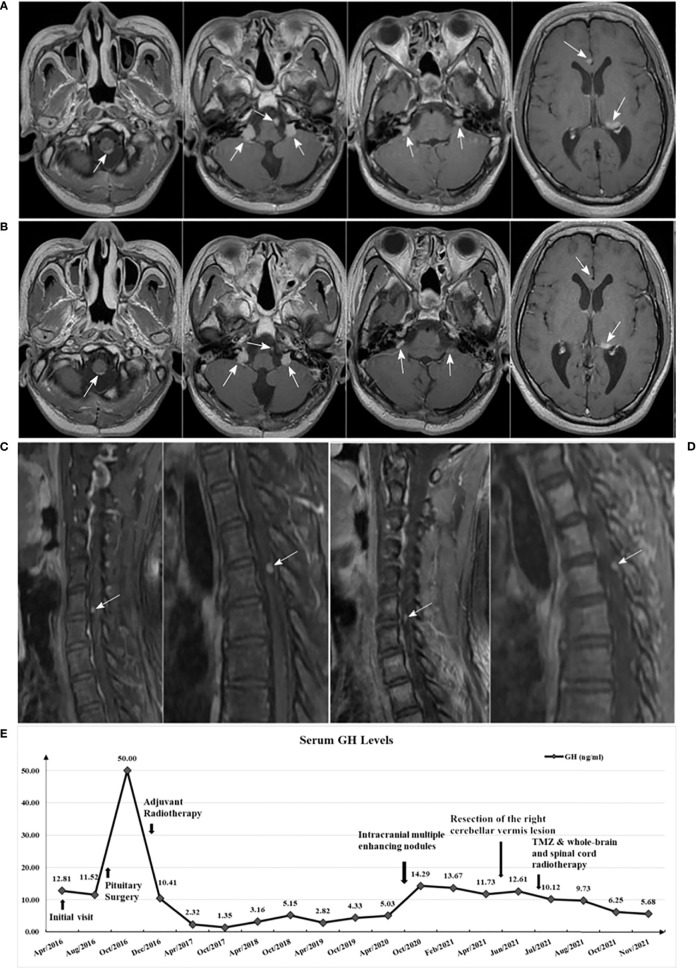
**(A)** Brain enhanced MRI in May 2021 (before treatment); **(B)** Brain enhanced MRI in November 2021 (after treatment) showed: multiple lesions all reduced in size compared with A; **(C)** Spinal cord enhanced MRI in June 2021 (before treatment); **(D)** Spinal cord enhanced MRI in November 2021 (after treatment) showed: enhanced nodules in the sixth cervical vertebrae and fourth thoracic vertebral plane of the spinal cord both mildly decreased compared with C; **(E)** Serum GH levels variation curve of the patient.

The patient underwent the combined treatment from June to August, 2021. After completion of radiotherapy, the patient didn’t stop TMZ treatment. With close monitoring, no side effects due to TMZ treatment were found. Follow-up MRI in November 2021 showed: metastases in both the intracranial and spinal cord appeared to shrink (**Figures 4A–D**), with reference to the RANO-BM criteria (7), the intracranial and spinal cord lesions were rated as partial response. In addition, the GH level decreased to 5.68 ng/mL (**Figure 4E**), and the IGF-1 level decreased to 378.0 μg/L.

## Literature review and discussions

The vast majority of PAs are benign and could be cured or controlled with surgery. However, a portion of tumors exhibit aggressive behavior (8). Like other well-differentiated neuroendocrine tumors, the incidence of IPAs is very low but higher than 0.2%. The degree of malignancy is also very low, but most of these aggressive tumors are histologically similar to malignancies with metastases (5). Pituitary adenohypophysial-cell tumors might invade and destroy the tissue in which they originate as well as adjacent tissues, and have a significant impact on patients’ health and quality of life (9). It should also be noted that “malignant” is not restricted to neoplasms that metastasize; it also applies to tumors that invade adjacent structures. Therefore, IPAs and PCs are two sides of the same coin, as mentioned by Trouillas et al. (4), who proposed to characterize IPAs as “tumors with malignant potential and no metastases.”

The diagnosis of PC is strictly limited to malignant tumors originating from adenohypophysial-cell, with the brain, spinal cord, and/or systemic metastases. Therefore, the early diagnosis of PC is extraordinarily difficult. Most PCs are functionally hormone-secreting, classified according to the secreted hormones (10). The etiology of PC remains unclear, and it may be associated with intraoperative dissemination of pituitary microadenomas and malignant progression of PAs. Early chromosomal mutations and the expression of proto-oncogenes may play an essential role in the occurrence and progression of PC (11). It has been investigated that an increase in chromosome 14q may be involved in the progression and malignant transformation of PAs (12, 13). The human oncogene p53 was found mutated in almost all patients with PC and 15% of patients with IPAs, but not in benign PAs (14). However, according to the survey by McCormack et al. (15), p53 expression did not differ between aggressive PT (APT) and PC.

GH-secreting PC is a complex and rare disease with no specific diagnostic markers to predict tumor metastasis and progression (16). Case reports of PCs published in recent 20 years were reviewed, of which only ten patients were GH-secreting PCs (17–25). The average age of all patients was 52.1 years, and male accounted for 60.0%. The most common sites of metastasis were cervical nodes and spinal cord, and the mean diagnosis time of metastasis was 13.0 years. Almost all the patients received surgery and radiotherapy, but only two patients received TMZ chemotherapy. However, the survival time of patients varied greatly. The details of the ten patients are shown in **Table 1**.

**Table 1:** Published cases of GH-secreting pituitary carcinomas in recent 20 years.

Authors and reference No.	Year	Sex	Age	Site of metastasis	Diagnosis time of metastasis (years)	Initial surgery	Radiotherapy	Chemotherapy	Survival time after diagnosis of pituitary carcinomas
le Roux et al. (17)	2001	F	47	Cervical nodes	2	+	+	−	Alive at the time of publication
Manahan et al. (18)	2007	F	68	Cervical nodes	30	+	+	−	Alive at the time of publication
Ilkhchoui et al. (19)	2010	M	31	Spinal cord	4	+	+	−	Not mentioned
Sreenan et al. (20)	2012	M	69	Cervical nodes	27	−	+	−	33 years
Lall et al. (21)	2013	M	66	Lateral orbit	14	+	+	−	Not mentioned
Tanaka et al. (22)	2013	M	60	Dural mater	13	+	+	−	1 year
Novruzov et al. (23)	2015	M	68	Intracranial and Spinal	20	+	+	−	4 years
Bengtsson et al. (24)	2015	F	40	Cerebral	11.6	+	+	TMZ	Alive at the time of publication
Bengtsson et al. (24)	2015	M	46	Intraspinal and Liver	2.7	+	+	TMZ	0.83 year
Wang et al. (25)	2015	F	26	Spinal cord	6	+	+	−	Alive at the time of publication

The 2017 edition of the World Health Organization (WHO) classification of PT defined SAs as adenomas that predominantly expressed GH and arose from cells of the Pit-1 spectrum (26). They were classified as densely granular SAs (DGSAs) and sparsely granular SAs (SGSAs). In contrast, SGSAs were more aggressive, had a high tendency to recur and were less manageable (27). Thus, for patients with pathological diagnosis of SGSAs, a standardized and individualized treatment plan should be formulated, and adjuvant treatment should be adopted after total or subtotal tumor resection with appropriate postoperative follow-up intervals, along with reasonable treatment modalities for early intervention in response to signs of recurrence.

Previous studies have shown that the Ki-67 index can be used to evaluate the mitotic status, which was of great significance for the diagnosis of PC (28). Some authors support that PTs with Ki-67 index more than 10% should be considered as primary PC (29). These claims may have some theoretical basis but lack validation of a large number of clinical data. However, despite more than 10 years of research, the significance of Ki-67 as a prognostic marker could not be established (30). In this case, the Ki-67 indexes of the patient in two surgeries were 2% and 8%, respectively (both <10%). If only based on the Ki-67 index, the patient would not be classified as a high-risk type. Thus, prognosis prediction of PTs must be based on a combined assessment of radiological manifestation, proliferation index and IHC, and any use of existing pathological features to predict PTs response to therapy, progression, and recurrence alone is not advisable.

The term “high-risk adenomas” was first introduced in the 4th edition of the WHO Classification of Tumors of Endocrine Organs in 2017 to define certain histological adenoma types with clinical aggressive characteristics, including sparsely granulated somatotroph, lactotroph in men, Crooke cell, silent corticotroph, and plurihormonal PIT-1 positive adenomas (31). The “high-risk adenomas” have a tendency for fast growth and frequent recurrences, which are difficult to control. Thus, it is of great significance to identify high-risk adenoma types early and accurately. Kontogeorgos et al. (32) recommended that in order to achieve precise histological classification, IHC for pituitary hormones and cytokeratins to identify high-risk adenoma types were required. Moreover, assessment of predictive markers by IHC played an important clinical role in pathology, contributing to achieving the maximum of the expected effectiveness of treatment.

The traditional treatment methods for PC included surgery, radiotherapy and chemotherapy, but for most patients with refractory PA and PC, the conventional treatments may not be effective, and TMZ may be a promising remedial therapy (15). According to the European Society of Endocrinology Clinical Practice Guidelines for the management of aggressive PTs and PCs (33), TMZ monotherapy was the first-line chemotherapy for aggressive PTs and PCs after the failure of standard therapies. Treatment evaluation after 3 cycles allowed identification of responder and non-responder patients. In patients responding to first-line TMZ, continuing treatment for at least 6 months in total was suggested. Moreover, the guideline recommended that patients with aggressive PTs should be managed by a multidisciplinary expert team, involving pathologists, radiologists, endocrinologists, and neurosurgeons. Such management could provide patients with more refined and individualized treatment recommendations. Moreover, for precise selection of patients to receive TMZ treatment, evaluation of O6-methylguanine-DNA methyltransferase (MGMT) expression by IHC using the appropriate antigen retrieval protocol, is strongly recommended (34).

After the treatment of TMZ combined with whole-brain and spinal cord radiotherapy, metastases in both the intracranial and spinal cord showed shrinkage, and the GH and IGF-1 level also decreased. The patient’s condition was controlled. To some extent, TMZ combined with radiotherapy may be a promising treatment for PC. With a lack of a large number of cases confirmations, this remains to be the direction of our follow-up studies. Meanwhile, whether TMZ should be used as early as possible to postpone the progression of high-risk PAs is a topic that needs to be investigated in the future.

## Conclusions

This rare case of sparsely granulated somatotroph carcinoma with cerebrospinal fluid dissemination showed promising signs of improvement on imaging, GH and IGF-1 tests after the treatment of TMZ combined with whole-brain and spinal cord radiotherapy, which indicated that this combination therapy was efficacious for the patient. We will conduct follow-up visits of the patient as well. Therefore, we would like to recommend: 1. Prognosis prediction of PTs must be based on a combined assessment of radiological manifestation, proliferation index and IHC; 2. Patients with IPA should be managed by a multidisciplinary expert team; 3. TMZ combined with radiotherapy may be a promising treatment for PCs.

## Data Availability Statement

The raw data supporting the conclusions of this article will be made available by the authors, without undue reservation.

## Ethics Statement

The studies involving human participants were reviewed and approved by Ethics Committee of Huashan Hospital, Fudan University. The patients/participants provided their written informed consent to participate in this study.

## Author Contributions

Conception and design: PD and XW. Collection and assembly of data: PD, XW, and JX. Manuscript writing and revising: All authors. All authors contributed to the article and approved the submitted version.

## Conflict of Interest

The authors declare that the research was conducted in the absence of any commercial or financial relationships that could be construed as a potential conflict of interest.

## Publisher’s Note

All claims expressed in this article are solely those of the authors and do not necessarily represent those of their affiliated organizations, or those of the publisher, the editors and the reviewers. Any product that may be evaluated in this article, or claim that may be made by its manufacturer, is not guaranteed or endorsed by the publisher.
